# Parametric Probability Distribution Functions for Axon Diameters of Corpus Callosum

**DOI:** 10.3389/fnana.2016.00059

**Published:** 2016-05-26

**Authors:** Farshid Sepehrband, Daniel C. Alexander, Kristi A. Clark, Nyoman D. Kurniawan, Zhengyi Yang, David C. Reutens

**Affiliations:** ^1^Centre for Advanced Imaging, The University of QueenslandBrisbane, QLD, Australia; ^2^Laboratory of Neuro Imaging, USC Mark and Mary Stevens Neuroimaging and Informatics Institute, Keck School of Medicine of USC, University of Southern CaliforniaLos Angeles, CA, USA; ^3^Department of Computer Science, Centre for Medical Image Computing, University College LondonLondon, UK; ^4^Brainnetome Center, Institute of Automation, Chinese Academy of SciencesBeijing, China; ^5^Faculty of Information Engineering, Southwest University of Science and TechnologyMianyang, China

**Keywords:** axon diameter distribution, probability distribution function, corpus callosum, electron microscopy, *generalized extreme value* distribution, gamma distribution

## Abstract

Axon diameter is an important neuroanatomical characteristic of the nervous system that alters in the course of neurological disorders such as multiple sclerosis. Axon diameters vary, even within a fiber bundle, and are not normally distributed. An accurate distribution function is therefore beneficial, either to describe axon diameters that are obtained from a direct measurement technique (e.g., microscopy), or to infer them indirectly (e.g., using diffusion-weighted MRI). The gamma distribution is a common choice for this purpose (particularly for the inferential approach) because it resembles the distribution profile of measured axon diameters which has been consistently shown to be non-negative and right-skewed. In this study we compared a wide range of parametric probability distribution functions against empirical data obtained from electron microscopy images. We observed that the gamma distribution fails to accurately describe the main characteristics of the axon diameter distribution, such as location and scale of the mode and the profile of distribution tails. We also found that the *generalized extreme value* distribution consistently fitted the measured distribution better than other distribution functions. This suggests that there may be distinct subpopulations of axons in the corpus callosum, each with their own distribution profiles. In addition, we observed that several other distributions outperformed the gamma distribution, yet had the same number of unknown parameters; these were the inverse Gaussian, log normal, log logistic and Birnbaum-Saunders distributions.

## Introduction

Axon diameter is an important structural characteristic of tissue in the central nervous system. Axon diameter correlates with conduction velocity, is affected by some neurological disorders, such as multiple sclerosis and autism, and changes during development (Ritchie, 1982; Piven et al., 1997; Bauman and Kemper, 2005; Hughes, 2006; Kunz et al., 2014). The conventional approach to obtain axon diameter values is through histological techniques such as electron microscopy (Aboitiz et al., 1992). Given the large number of axons in a region of interest and the variation in axon diameter, a statistical representation such the distribution of axon diameters distribution, is useful to describe axon diameter. In additional, recent efforts to infer axon diameter non-invasively using diffusion-weighted MRI often utilize a statistical model of axon diameter distribution, to be fitted to MRI measurements (Stanisz et al., 1996; Assaf et al., 2008; Barazany et al., 2009; Alexander et al., 2010; Dyrby et al., 2013; McNab et al., 2013; Horowitz et al., 2014; Huang et al., 2015; Sepehrband et al., 2016).

The gamma distribution is the most common probability distribution function used for this purpose. It has two parameters, shape and scale, and broadly reflects the shape of the axon diameter distribution, which has been consistently observed to be right-skewed and heavy-tailed (i.e., an asymmetric distribution, in which the right tail of the distribution is much longer than the left tail). Recently Pajevic and Basser (2013) argued, from neurophysiological perspective, that this skewed profile optimizes information transfer and capacity along bundles of axons. They also reported optimum distributions, based on parameters describing the fiber's ability to transmit information, that outperform the gamma distribution, in practical applications such as AxCaliber (Assaf et al., 2008).

Here we introduce another probability distribution function that provides a good representation of the heavy-tailed axon diameter distribution, the *generalized extreme value* distribution. We empirically compared the generalized extreme value distribution with the gamma distribution and fourteen others, without any prior assumptions with respect to the anatomy or metabolic requirements of axons. We assessed different probability functions using electron microscopy images of mouse corpus callosum in which we manually measured more than 20,000 axons. In addition, we examined previously published electron microscopy data for the human and macaque corpus callosum (Liewald et al., 2014).

## Materials and methods

**Figure 1:**
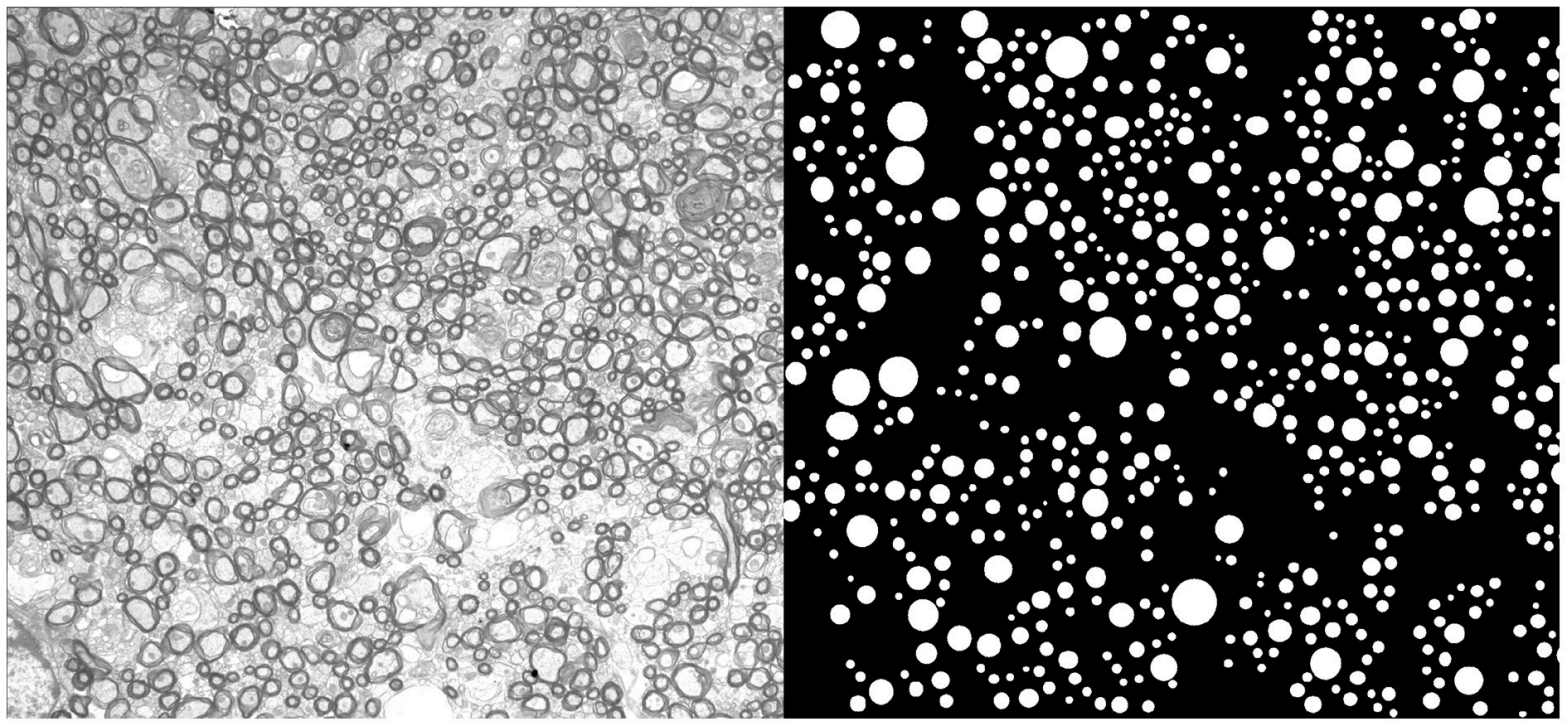
**An example electron microscopy image of mouse corpus callosum**. On the right panel the circular representation of the measured axons is presented.

Axons of the corpus callosum of a mouse corpus callosum were manually measured using cross sectional electron microscopy images. Animal preparation and electron microscopy are described in detail in (Sepehrband et al., 2016), but are also included in Appendix A for readability. Electron microscopy images and measurements are available at: https://github.com/sepehrband/AxonDiameter. Figure 1 shows a representative electron microscopy image and measured axons. In addition, we investigated the axon diameter distribution profile of human and macaque from an electron microcopy study performed by Liewald et al. (2014). Three regions of two human corpus callosum (genu, truncus, and splenium) and a region of macaque corpus callosum (truncus) were examined; from Figure 10 of Liewald et al. (2014).

Sixteen different parametric continuous probability distribution functions were fitted to the axon diameter data (Table 1; Severini, 2005). In particular, distribution functions with three or fewer unknown parameters were used. The beta distribution was excluded from the comparison, as it only defines the values in 0 to 1 range, and even after normalizing axon diameter values to this range, it fitted the data poorly (see Supplementary Figure 1). Distribution functions were fitted using the *allfitdist* function, in the MATLAB® statistics toolbox.

**Table 1:** **Investigated probability distribution function, their parameters and their mathematical functions**.

**Name**	**Parameters**	**Probability density function**
Birnbaum- Saunders	Scale (β) Shape (γ)	$f\left(x;\beta ,\gamma \right)=\frac{\sqrt{\frac{x}{\beta }}+\sqrt{\frac{\beta }{x}}}{2\gamma \left(x\right)}\phi \left(\frac{\sqrt{\frac{x}{\beta }}-\sqrt{\frac{\beta }{x}}}{\gamma }\right)$
Exponential	Mean (λ)	$f(x;\lambda )=\{\begin{array}{cc} \lambda e^{-\lambda x} & x\geq 0,\\ 0 & x<0. \end{array}$
Extreme value	Location (μ) Scale (σ)	$f\left(x;\mu ,\sigma \right)=\frac{1}{\sigma }\exp\left[-\left(\frac{x-\mu }{\sigma }\right)\right]\exp\left\{-\exp\left[-\left(\frac{x-\mu }{\sigma }\right)\right]\right\}$
Gamma	Shape (κ) Scale (θ)	$f\left(x;k,\theta \right)=\frac{x^{k-1}e^{-\frac{x}{\theta }}}{\theta^{k}\Gamma \left(k\right)}$
Generalized extreme value	Location (μ) Scale (σ) Shape (ξ)	$f\left(x;\mu ,\sigma ,\xi \right)=\frac{1}{\sigma }+{\left[1+\xi \left(\frac{x-\mu }{\sigma }\right)\right]}^{\left(-1∕\xi \right)-1}\exp\left\{-{\left[1+\xi \left(\frac{x-\mu }{\sigma }\right)\right]}^{-1∕\xi }\right\}$
Generalized Pareto	Shape (ξ) Threshold (μ) Scale (σ)	$f\left(x;\xi ,\mu ,\sigma \right)=\frac{1}{\sigma }{\left(1+\frac{\xi \left(x-\mu \right)}{\sigma }\right)}^{\left(-\frac{1}{\xi }-1\right)}$
Inverse Gaussian	Scale (μ) Shape (λ)	$f\left(x;\mu ,\lambda \right)={\left[\frac{\lambda }{2\pi x^{3}}\right]}^{1∕2}\exp{\frac{-\lambda \left(x-\mu \right)}{2\mu^{2}x}}^{2}$
Log logistic	Log scale (α) Log location (β)	$f\left(x;\alpha ,\beta \right)=\frac{\left(\beta ∕\alpha \right){\left(x∕\alpha \right)}^{\beta -1}}{{\left(1+{\left(x∕\alpha \right)}^{\beta }\right)}^{2}}$
Log normal	Log location (μ) Log scale (β)	$f\left(x;\mu ,\sigma \right)=\frac{1}{x\sigma \sqrt{2\pi }}e^{-\frac{{\left(\ln\;x-\mu \right)}^{2}}{2\sigma^{2}}}$
Logistic	Location (μ) Scale (*s*)	$f\left(x;\mu ,s\right)=\frac{e^{-\frac{x-\mu }{s}}}{s{\left(1+e^{-\frac{x-\mu }{s}}\right)}^{2}}$
Nakagami	Shape (*m*) Spread (Ω)	$f\left(x;m,\Omega \right)=\frac{2m^{m}}{\Gamma \left(m\right)\Omega^{m}}x^{2m-1}\exp\left(-\frac{m}{\Omega }x^{2}\right)$
Normal	Location (μ) Scale (√σ)	$f\left(x,\mu ,\sigma \right)=\frac{1}{\sigma \sqrt{2\pi }}e^{-\frac{{\left(x-\mu \right)}^{2}}{2\sigma^{2}}}$
Rayleigh	Scale (σ)	$f\left(x;\sigma \right)=\frac{x}{\sigma^{2}}e^{-x^{2}∕\left(2\sigma^{2}\right)}$
Rician	Nocentrality (ν) Scale (σ)	$f\left(x;\nu ,\sigma \right)=\frac{x}{\sigma^{2}}\exp\left(\frac{-\left(x^{2}+\nu^{2}\right)}{2\sigma^{2}}\right)I_{0}\left(\frac{x\nu }{\sigma^{2}}\right)$
t location-scale	Degree of freedom (ν) Scale (σ) Location (μ)	$f\left(x;\nu ,\sigma ,\mu \right)=\frac{\Gamma \left(\frac{\nu +1}{2}\right)}{\sigma \sqrt{\nu \pi \Gamma \left(\nu 2\right)}}{\left(1+\frac{\frac{{\left(x-\mu \right)}^{2}}{\sigma }}{\nu }\right)}^{-\frac{\nu +1}{2}}$
Weibull	Scale (λ) Shape (κ)	$f\left(x;\lambda ,k\right)=\{\begin{array}{cc} \frac{k}{\lambda }{\left(\frac{x}{\lambda }\right)}^{k-1}e^{-{\left(x/\lambda \right)}^{k}} & x\geq 0,\\ 0 & x<0, \end{array}$

To evaluate the performance of the distribution functions, the Akaike Information Criterion (AIC) was used (Akaike, 1974); it trades off goodness of fit against model complexity. AIC measures the relative quality of a statistical model, in which the model with the lowest AIC score is ranked highest. The AIC was corrected for finite sample sizes and was calculated as follows:

$$\begin{array}{l} AIC=2k-2\text{ln}\left(\text{L}\right)+\frac{2k\left(k+1\right)}{n-k-1}, \end{array}$$

where *L* and *k* are the maximum value of the likelihood function and the number of estimated parameters, respectively. In addition to AIC, the Bayesian Information Criterion (BIC) amd negative log-likelihood values were also assessed. However, we only report the AIC, as all criteria used led to same rankings of tested distributions.

## Results

### Basic statistics

Mean axon diameters were around 0.56 μm in different regions of the corpus callosum (Table 2). The largest axons were observed in the body and genu, and the smallest axons in the splenium. The mean axon diameter in the genu was significantly smaller than in both the body and the splenium (*p* < 0.01). The mean axon diameter in the splenium was only slightly smaller than in the body, but the difference was not significant. The splenium has the highest median value and the lowest standard deviation, demonstrating homogeneity of axon diameter in this region. A similar trend was seen in other mammalian species (Olivares et al., 2001).

**Table 2:** **Basic statistics of axon diameters of the mouse corpus callosum, obtained from electron microscopy**.

**Region**	***N***	**Mean ± *S.D*. (μm)**	**Min.— Max. (μm)**	**Median (μm)**
Genu	7680	0.54 ± 0.28	0.14 − 3.09	0.47
Body	5260	0.57 ± 0.29	0.16 − 2.76	0.49
Splenium	7188	0.57 ± 0.23	0.03 − 2.26	0.52
Whole CC	20128	0.56 ± 0.27	0.03 − 3.09	0.49

### Axon diameter distribution in the corpus callosum

Table 3 shows the ranking of the evaluated probability distribution functions. Regardless of the evaluation criterion, the same ranking was obtained. The generalized extreme value distribution, while having three unknown parameters, ranked highest. Log normal, inverse Gaussian, log logistic, and Birnbaum-Saunders distributions, with relatively similar AIC values and the same number of unknown parameters, were good alternatives to represent the distribution of axon diameter. The gamma distribution, despite also having two unknown parameters, performed relatively poorly. The Generalized Pareto and t-location-scale distributions performed poorly, even though they both have three unknown parameters. As expected, the normal distribution was also ranked low; it fails to represent the skewness of axon diameter distribution. Interestingly, while the generalized extreme value distribution outperformed other distributions, the extreme value distribution had the lowest ranking. Extreme value distribution models extreme deviation from the median of probability distribution, but may fail to accurately describe the rest of the distribution. Generalized extreme value distribution, however, combines three types of extreme value distributions, allowing a continuous range of possible shapes, which most likely explains the divergence of performance.

**Table 3:** **Ranking of different distribution functions, used to describe axon diameter distribution of mouse corpus callosum**.

**Distribution function**	**Rank**	**Parameters**	**AIC**
Generalized extreme value	1	3	−6159
Log normal	2	2	−5411
Inverse gaussian	3	2	−5367
Log logistic	4	2	−5360
Birnbaum-saunders	5	2	−5252
Gamma	6	2	−3463
*t* location-scale	7	3	−1147
Nakagami	8	2	−273
Logistic	9	2	498
Weibull	10	2	773
Rayleigh	11	1	1038
Rician	12	2	1040
Normal	13	2	4385
Generalized pareto	14	3	12182
Exponential	15	1	16756
Extreme value	16	2	21256

*AIC, Akaike Information Criterion*.

**Figure 2:**
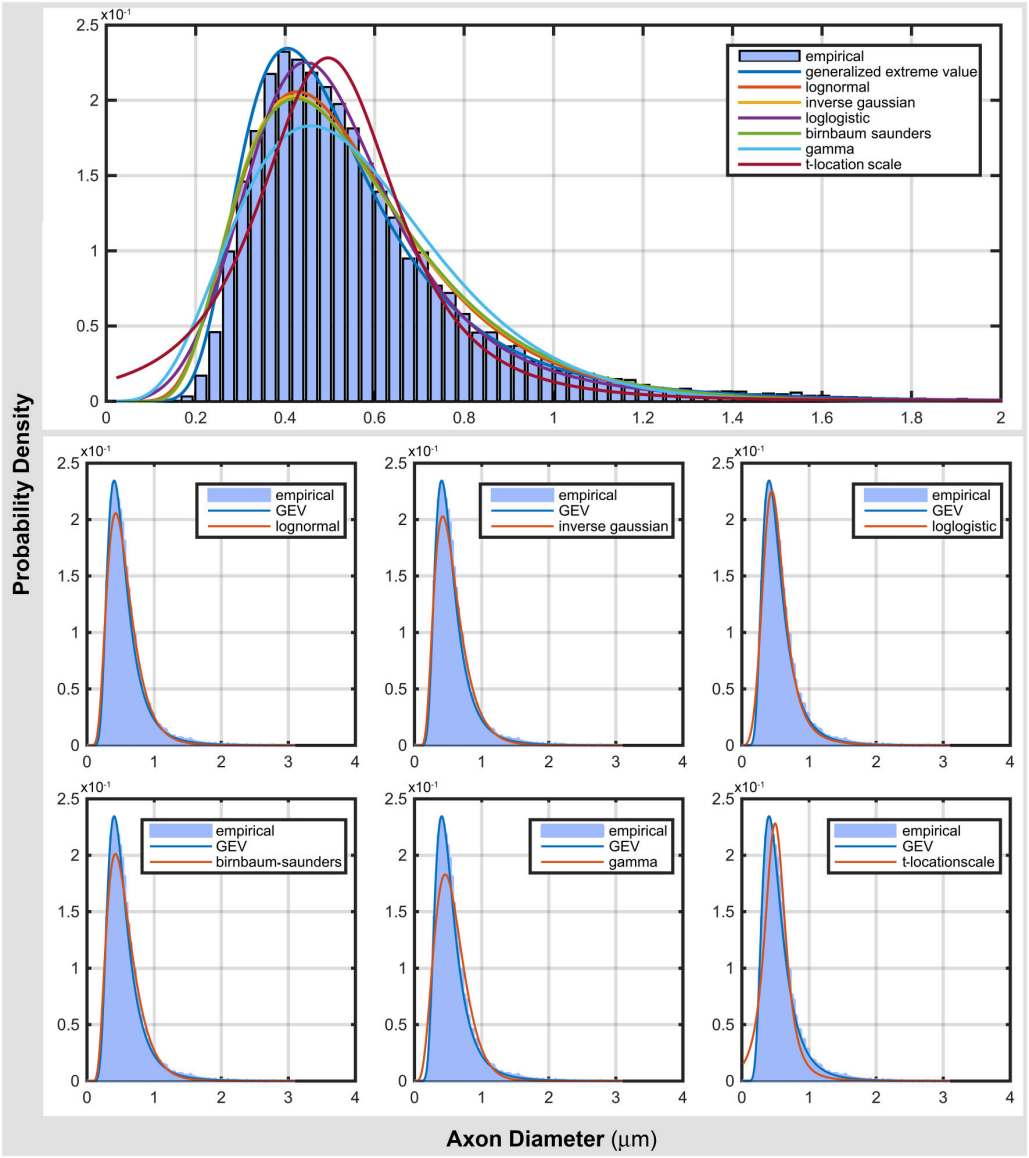
**Comparing top ranked probability density functions with empirical data from electron microscopy**.

Figure 2 compares the top seven probability distribution functions with the empirical data. As shown in Table 3, generalized extreme value distribution gave an accurate representation of the data. Most of distribution functions accurately represented the location of the mode (peak of the distribution), but failed to represent the scale of the mode (see log normal, inverse Gaussian, and Birnbaum-Saunders). The gamma and t-location-scale distributions missed both location and scale of the mode. In addition, they gave a poor representation of both tails of the distribution. These poor representations raise questions about the reliability of the neuroanatomical measures obtained from the commonly used gamma distribution.

**Figure 3:**
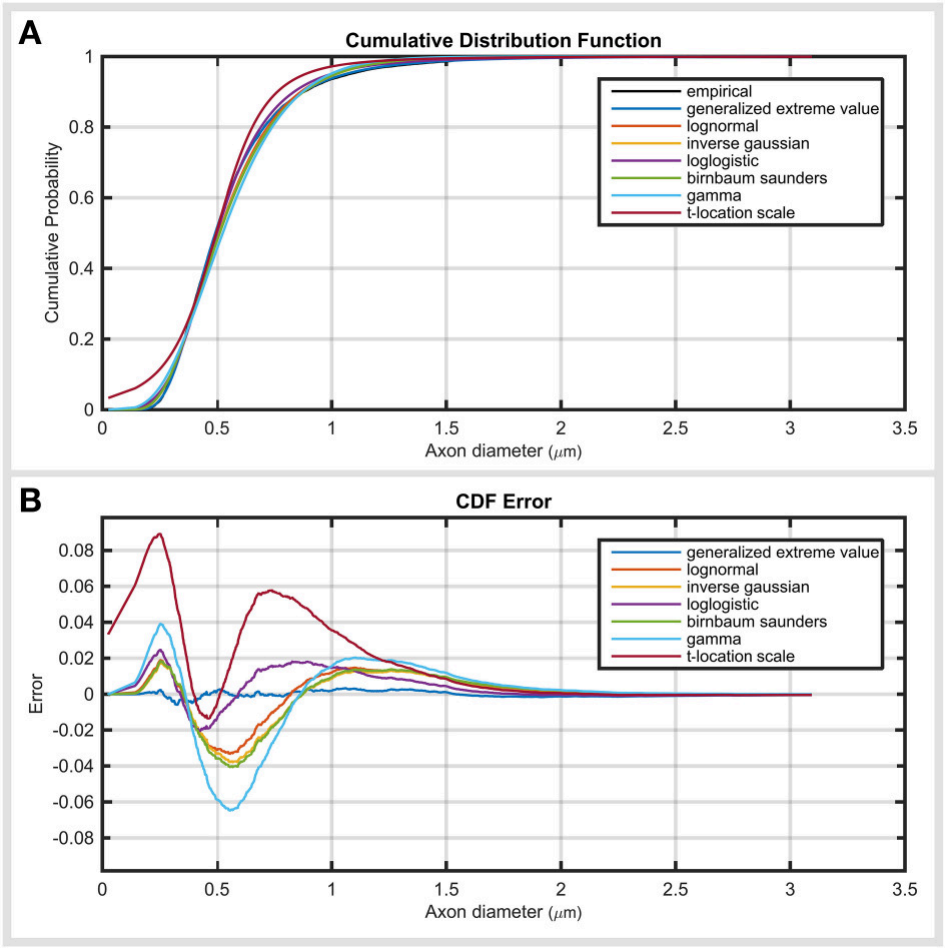
**Investigating the error propagation across the data. (A)** Cumulative distribution function of top ranked distribution functions compared with empirical data. **(B)** Error of cumulative distribution functions throughout the axon diameter values. Error values demonstrate the amount and location of the under- and over-estimation of the distribution of the cumulative distribution compared to the empirical data.

Figure 3 demonstrates the cumulative distribution of top five distribution functions, together with their error. The error plot (Figure 3B) shows that generalized extreme value function had relatively constant errors across axon diameter values. The remaining distributions showed two error peaks, one before and one after the mode. gamma and *t*-location-scale distributions had the highest negative and positive error values.

### Sub-regions of the corpus callosum

**Figure 4:**
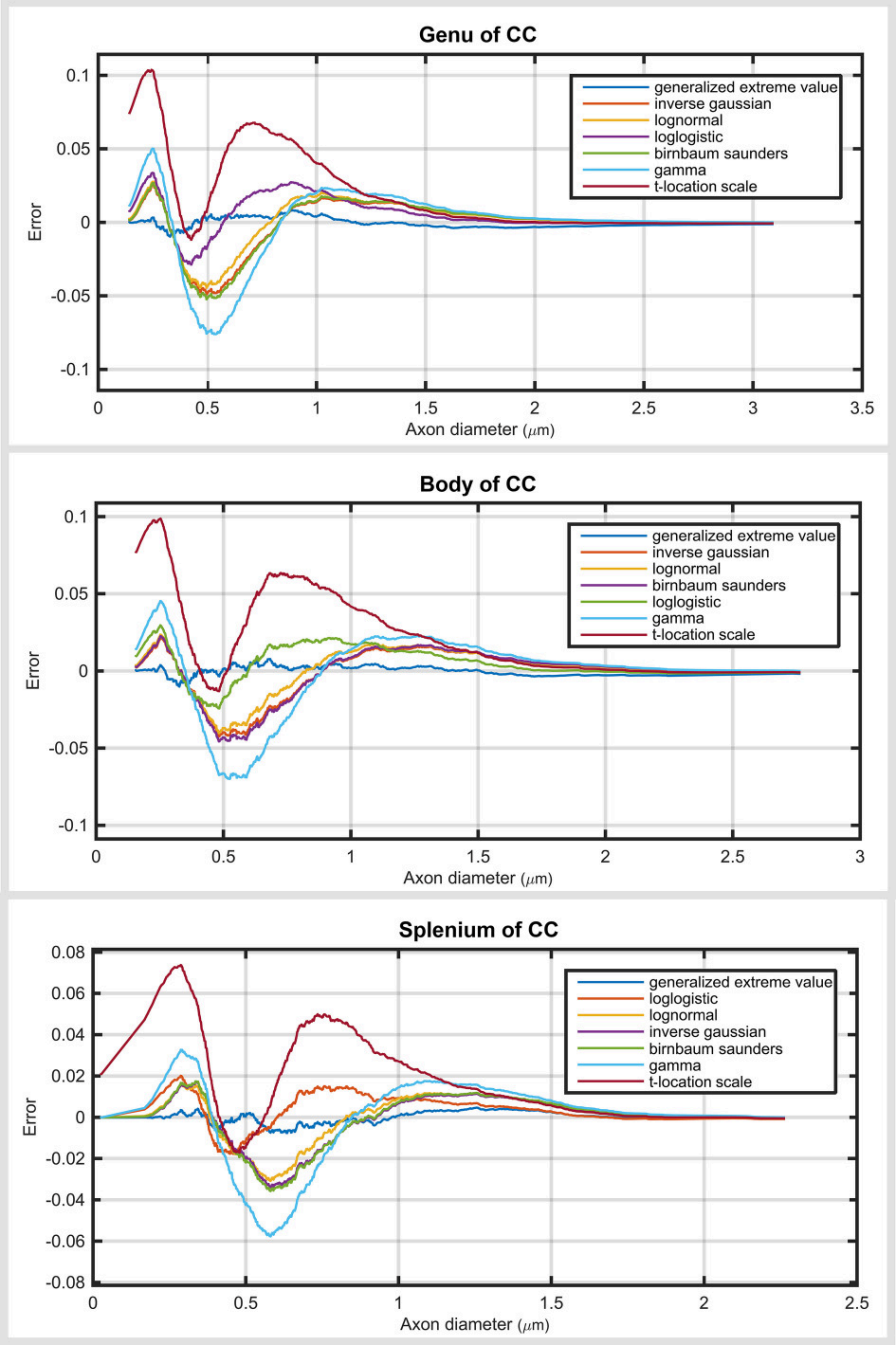
**Plots are the cumulative distribution error for three sub-regions of the corpus callosum: genu, body and splenium for seven top ranked probability distribution functions**.

The main difference between the sub-regions was in skewness (Figure 4). The axon diameter distribution of the genu and body was more skewed compared with that of the splenium. Regardless of region, the generalized extreme value distribution always ranked highest. As expected, the ranking of the probability distribution functions in the genu and body (with relatively similar distribution profile) was almost the same as for the splenium. In the splenium, log logistic and log normal distributions were ranked second and third, respectively. Regardless of the region, gamma and *t*-location-scale distributions were the lowest ranked of the top seven. Similar to the analysis of the whole corpus callosum, the gamma distribution had high negative and positive error values compared with other top ranked distribution functions. Supplementary Tables show the ranking of all the distribution functions across sub-regions of the corpus callosum.

Corroborating our mouse brain data, the generalized extreme value distribution consistently ranked highest when human or monkey corpus callosum were assessed (Table 4). Similar to previous results, the gamma distribution ranked sixth, regardless of the region or sample. The inverse Gaussian, log normal, log logistic, and Birnbaum-Saunders distributions were ranked 2nd to 5th, with the inverse Gaussian appearing to give a slightly better fit to the data. A further non-parametric Friedman's test followed by *post-hoc* Nemenyi test was performed on the ranking results of the top six probability distribution functions to assess whether the goodness of fit of different probability functions differed significantly (Supplementary Figure 2). The probability distribution function affected goodness of fit significantly (*p* < 0.0001). The gamma distribution was significantly poorer fitting than the generalized extreme value, inverse Gaussian, log normal and log logistic distributions (*p* < 0.05).

**Table 4:** **Ranking of top six probability distribution functions across regions of human and monkey corpus callosum; axon diameter distributions were borrowed from electron microcopy study of Liewald et al. (2014)**.

**Probability distribution function**	**Human 1**			**Human 2**			**Monkey**
	**cc1**	**cc2**	**cc3**	**cc1**	**cc2**	**cc3**	**cc2**
Generalize extreme value	1	1	1	1	1	1	1
Inverse gaussian	2	3	2	2	2	2	4
Log normal	3	4	3	4	3	4	3
Log logistic	4	2	4	5	5	3	2
Birnbaum-saunders	5	5	5	3	4	5	5
Gamma	6	6	6	6	6	6	6

*cc1, cc2, and cc3 are genu, truncus, and splenium, respectively*.

## Discussion

In this work, we investigated the optimum probability distribution function for describing axons of corpus callosum. The optimum probability distribution is most useful for techniques that use mathematical models either to describe axon diameters that are measured directly (e.g., from electron histology), or to infer the distribution of axon diameter from non-invasive measurements indirectly (e.g., diffusion-weighted MRI). To find the optimum probability distribution function, we performed electron microscopy of the corpus callosum of an adult C57Bl/6J mouse and carefully measured the diameter of more than 20,000 axons. We also assessed electron microscopy data for human and macaque corpus callosum from the literature. Model selection for axon diameter distribution functions was based on information criteria (i.e., AIC) and error propagation.

Gamma distribution failed to accurately describe the main characteristics of the axon diameter distribution, such as location and scale of the mode and the profile of distribution tails. On the contrary, generalized extreme value distribution consistently fitted the measured distribution better than other distribution functions. Axon morphology correlates with axonal function (e.g., axon diameter correlates with conduction velocity). It is possible that axons fall into different subpopulations with different distribution profiles; i.e., axons with small diameter may have a different distribution profile to large diameter axons. Therefore, a distribution function that can capture such a characteristic would outperform others. The generalized extreme value combines three simpler distributions and may fit better because it can capture different subpopulations simultaneously.

Techniques such as AxCaliber that use a mathematical model of tissue microstructure to indirectly infer axon diameter distribution require a small number of unknown parameters. The generalized extreme value distribution function has one more unknown parameter than the gamma distribution. Four other probability distribution functions outperformed the gamma distribution and, like the gamma distribution, also have only two unknown parameters (Tables 1, 4). Log-normal, log-logistic, and inverse Gaussian distribution functions proved to be significantly better descriptors of axon diameter distribution than the gamma distribution function. In particular, the log-normal model which outperforms the gamma distribution in all of our comparisons, has the virtues of simplicity, widespread use in biology and neurobiological validity (Buzsáki and Mizuseki, 2014).

Unlike previous studies (Gov, 2009; Perge et al., 2012; Pajevic and Basser, 2013), we did not explicitly focus on explaining the skewness of the axon diameter distribution. Rather, we evaluated a range of parametric probability distribution functions to find the most parsimonious model in terms of unknown parameters that optimized model accuracy. Model selection did not consider prior knowledge about axon morphometry.

As discussed in (Sepehrband et al., 2016), our estimated values are higher than those reported in a previous study (median of 0.25 μm and mean of 0.43 μm) (Innocenti et al., 1995). The difference could be due to the shrinkage artifact caused by older techniques for embedding and fixation of the tissue. We used a method that, in some settings, has been demonstrated to produce almost no shrinkage during processing compared to 40–70% shrinkage with other techniques (Hanssen et al., 2013).

## Author contributions

FS, DA, KC, ZY, and DR designed the experiment. FS, NK, collected the data. FS measured axon diameters and performed statistical analysis. FS wrote the manuscript. All authors contributed to the manuscript.

## Funding

FS was supported by a University of Queensland Centennial (UQCent) and International Postgraduate Research Scholarship (IPRS). DR was supported by National Health and Medical Research Council of Australia (Program Grant 628952). EPSRC grants G007748 I027084 L022680 and M020533 support DA's work on this topic. KC was supported by the National Institute of Health Grants R00HD065832, R01MH094343, P41EB015922, and U54EB020406.

### Conflict of interest statement

The authors declare that the research was conducted in the absence of any commercial or financial relationships that could be construed as a potential conflict of interest.

## Appendix A

### Animal housing and preparation

All procedures were performed with the approval of The University of Queensland Animal Ethics Committee, under the guidelines of the National Health and Medical Research Council of Australia. One 8-week-old male C57Bl/6J mouse was reared and housed at the Queensland Brain Institute animal facility, The University of Queensland. The animal was housed on a 12 h light/dark cycle and free access to food and water were provided.

We scanned a mouse brain, as mouse models of neurological diseases are commonly used due to the availability of relevant mouse mutants and of gene targeting technology (Sillitoe et al., 2012). The adult mouse was anesthetised with an intraperitoneal injection of approximately 8–9 mg/mL sodium pentobarbitone (Lethabarb™; Virbac, AU) and then transcardially perfused with 0.9% saline solution (0.9% w/v NaCl in MilliQ™ (Millipore, AU) water) for 5 min followed by 4% PFA (4% w/v paraformaldehyde; ProSciTech, AU) with 0.2% Magnevist® (1 mM gadopentetate dimeglumine, Bayer) in PBS (phosphate-buffered saline, 137mM NaCl; 10mM Na2HPO4; 1.8mM KH2PO4; 2.7mM KCl; pH 7.4) for 10 min. The steps for fixation and storage are similar to Dyrby et al. (2011), previously optimized for diffusion-weighted MRI studies. The brain was post-fixed in 4% PFA with 0.2% Magnevist® in PBS and stored at 4°C. The brain was then incubated in PBS and 0.2% Magnevist for 4 days prior to MRI to remove the PFA prior to MRI. A Magnevist concentration of 0.2% and incubation for 4 days were found to be optimal for obtaining good SNR and contrast for *ex vivo* brain imaging (Huang et al., 2009). A high concentration of Magnevist can influence estimation of extra-axonal water diffusivity (D'Arceuil et al., 2007) and fraction but at the concentration used in this study, Magnevist does not affect the diffusion properties such as apparent diffusion coefficient and fractional anisotropy (D'Arceuil et al., 2007). The calculated water: Magnevist ratio in the white matter is around 1/5500 with a very short interaction of 1 ns between two molecules and radius of separation between the two molecules being 0.1–1 nm (Micskei et al., 1993; Sherry et al., 2009).

### Histological imaging

The brain was sectioned sagittally at 50 μm thickness using a vibratome after the imaging. The sections were placed in 12 well-plates containing PBS with sodium azide and stored at 4°C. A mid-sagittal section was selected. EM imaging was then performed on sub-sections of the corpus callosum.

On the mid-sagittal section, the corpus callosum was isolated and samples of the genu, body, and splenium were separated. Sample preparation was carried out according to the methods of Wilke et al. (2013). After polymerization of the resin blocks, sections were cut on a UC6 ultra-microtome (ultracut S, Reichert, Leica, Sweden) at 60 nm, and imaged at x5000 in a transmission electron microscope at 80 kV (JEM 1011, Jeol, Japan) at the Centre for Microscopy and Microanalysis of The University of Queensland. Images were captured with an Olympus Morada digital camera.

Estimation of axon diameters from EM was performed in MATLAB® software, version R2013a (Mathworks, Natick, MA). Each axon was manually selected by drawing a circle on its transverse section (20,128 axons were segmented). Basic statistics of the axon diameters were then calculated. One-way Analysis of Variance (ANOVA) with Tukey-Kramer *post-hoc* correction was performed to evaluate mean differences across sub-regions of the corpus callosum. *P* < 0.01 was taken to be statistically significant.
