# Supplementary material for: Parametric Probability Distribution Functions for Axon Diameters of Corpus Callosum
Source: Front Neuroanat. 2016 May 26;10:59. doi: 10.3389/fnana.2016.00059 (PMC4880597; doi:10.3389/fnana.2016.00059)
Supplement: Supplementary file 1 [file Table1.DOCX]

Supplementary Material

Parametric Probability Distribution Functions for Axon Diameters of Corpus Callosum

Farshid Sepehrband, Daniel C. Alexander, Kristi A. Clark, Nyoman D. Kurniawan, Zhengyi Yang, David C. Reutens

*** Correspondence:**

Farshid Sepehrband
farshid.sepehrband@loni.usc.edu

# Supplementary Tables

## Genu of the corpus callosum

**Table 1.** Ranking of parametric distribution functions to describe axon diameters of the genu of mouse corpus callosum

| Distribution function | Rank | Parameters | AIC | BIC |
| --- | --- | --- | --- | --- |
| Generalized Extreme Value | 1 | 3 | -2328 | -2349 |
| Inverse Gaussian | 2 | 2 | -1900 | -1914 |
| Log normal | 3 | 2 | -1883 | -1897 |
| Log logistic | 4 | 2 | -1842 | -1856 |
| Birnbaum Saunders | 5 | 2 | -1837 | -1851 |
| Gamma | 6 | 2 | -978 | -992 |
| t-Location-Scale | 7 | 3 | 7 | -14 |
| Nakagami | 8 | 2 | 410 | 396 |
| Weibull | 9 | 2 | 636 | 622 |
| Rayleigh | 10 | 1 | 633 | 626 |
| Rician | 11 | 2 | 642 | 628 |
| Logistic | 12 | 2 | 813 | 800 |
| Generalized pareto | 13 | 3 | 933 | 912 |
| Normal | 14 | 2 | 2439 | 2425 |
| Exponential | 15 | 1 | 5988 | 5981 |
| Extreme value | 16 | 2 | 9146 | 9132 |
| AIC: Akaike Information Criterion  BIC: Bayesian Information Criterion | |  |  |  |

## Body of the corpus callosum

**Table 2.** Ranking of parametric distribution functions to describe axon diameters of the body of mouse corpus callosum

| Distribution function | Rank | Parameters | AIC | BIC |
| --- | --- | --- | --- | --- |
| Generalized Extreme Value | 1 | 3 | -933 | -953 |
| Inverse Gaussian | 2 | 2 | -736 | -749 |
| Log normal | 3 | 2 | -721 | -735 |
| Birnbaum Saunders | 4 | 2 | -698 | -711 |
| Log logistic | 5 | 2 | -676 | -690 |
| Gamma | 6 | 2 | -163 | -176 |
| t-Location-Scale | 7 | 3 | 566 | 546 |
| Nakagami | 8 | 2 | 714 | 701 |
| Weibull | 9 | 2 | 867 | 854 |
| Rayleigh | 10 | 1 | 866 | 860 |
| Rician | 11 | 2 | 875 | 862 |
| Generalized pareto | 12 | 3 | 883 | 864 |
| Logistic | 13 | 2 | 1041 | 1028 |
| Normal | 14 | 2 | 2048 | 2035 |
| Exponential | 15 | 1 | 4554 | 4547 |
| Extreme value | 16 | 2 | 6167 | 6154 |
| AIC: Akaike Information Criterion  BIC: Bayesian Information Criterion | |  |  |  |

## Splenium of the corpus callosum

**Table 3.** Ranking of parametric distribution functions to describe axon diameters of the splenium of mouse corpus callosum

| Distribution function | Rank | Parameters | AIC | BIC |
| --- | --- | --- | --- | --- |
| Generalized Extreme Value | 1 | 3 | -3373 | -3393 |
| Log logistic | 2 | 2 | -3215 | -3228 |
| Log normal | 3 | 2 | -3175 | -3188 |
| Inverse Gaussian | 4 | 2 | -3096 | -3110 |
| Birnbaum Saunders | 5 | 2 | -3075 | -3088 |
| Gamma | 6 | 2 | -2663 | -2677 |
| t-Location-Scale | 7 | 3 | -2008 | -2029 |
| Nakagami | 8 | 2 | -1759 | -1773 |
| Logistic | 9 | 2 | -1630 | -1644 |
| Weibull | 10 | 2 | -1069 | -1083 |
| Rician | 11 | 2 | -863 | -877 |
| Normal | 12 | 2 | -477 | -491 |
| Rayleigh | 13 | 1 | -465 | -471 |
| Generalized pareto | 14 | 3 | 4034 | 4014 |
| Extreme value | 15 | 2 | 4951 | 4937 |
| Exponential | 16 | 1 | 6229 | 6222 |
| AIC: Akaike Information Criterion  BIC: Bayesian Information Criterion | |  |  |  |

# Supplementary Figures

**
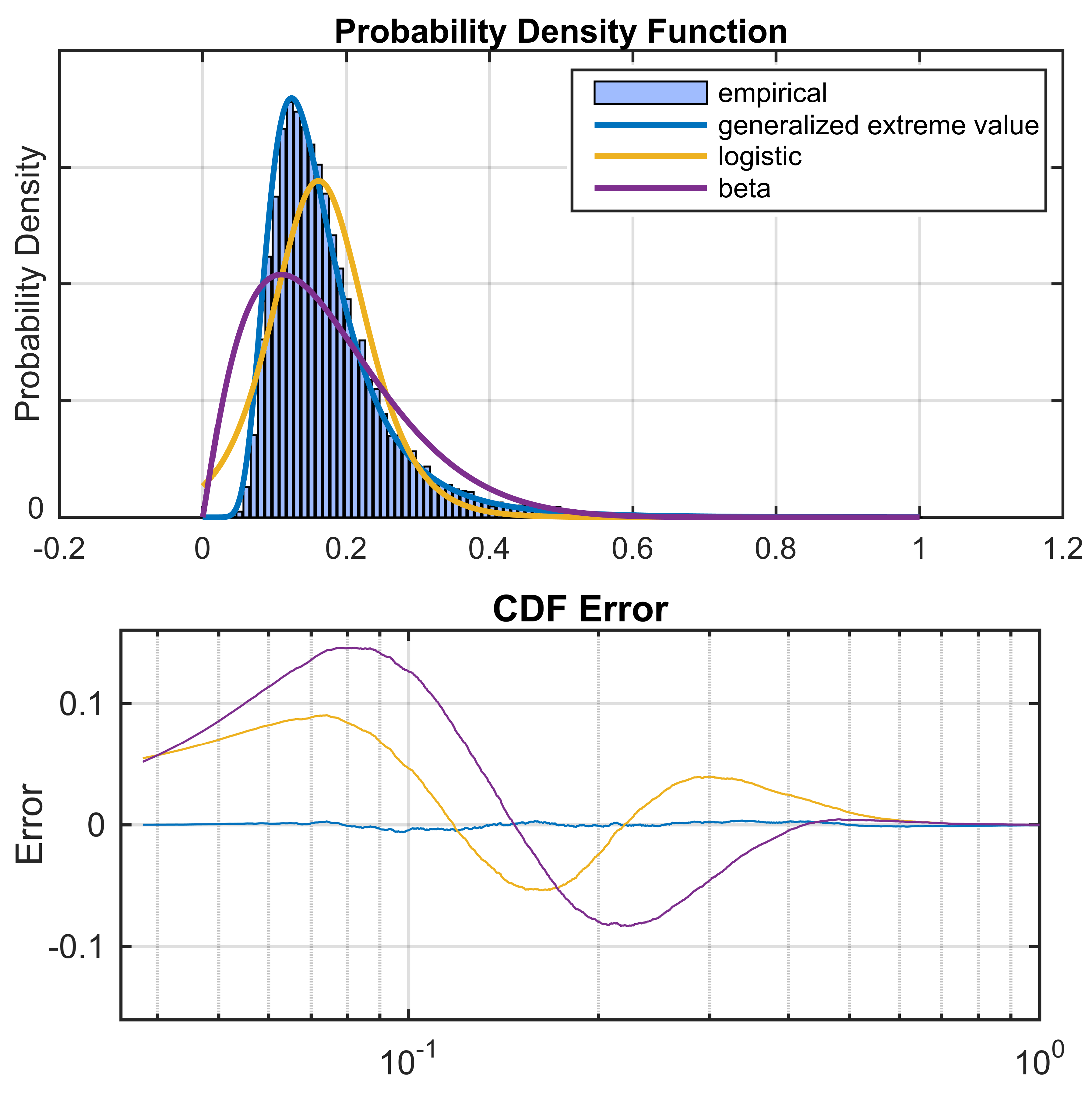
**

**Supplementary Figure 1.** Comparing Beta distribution with empirical data from electron microscopy. Generalized extreme value (top ranked distribution) and logistic distribution (ranked 9^th^ out of sixteen) are also included for purposes of comparison. The upper plot evaluates the Beta probability distribution function and the lower plot demonstrates the error of cumulative distribution functions. Note that axon diameter values were normalized prior to model assessment.


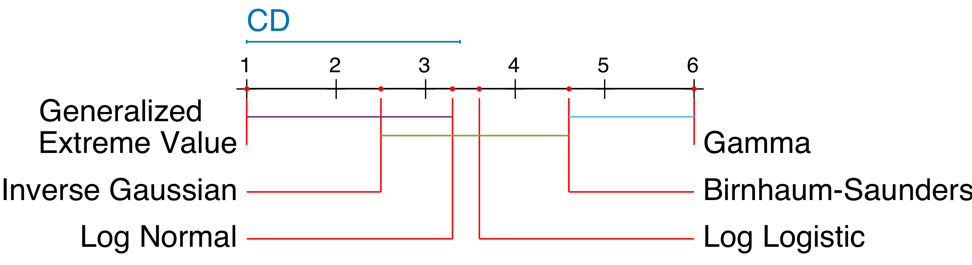


**Supplementary Figure 2.** A non-parametric Friedman’s test was used to assess whether the choice of probability distribution function had a significant effect on goodness-of-fit (*p*=5.4e-08). Probability distribution functions that do not differ significantly according to the post hoc Nemenyi test (*p*-value of 0.05) are connected by a horizontal line. CD is the critical distance. Statistical test was performed using the *drawNemenyi* function using MATLAB R2015b, as described in: <https://github.com/sepehrband/drawNemenyi>.
